# The Impact of Pre-existing Comorbidities and Therapeutic Interventions on COVID-19

**DOI:** 10.3389/fimmu.2020.01991

**Published:** 2020-08-11

**Authors:** Lauren A. Callender, Michelle Curran, Stephanie M. Bates, Maelle Mairesse, Julia Weigandt, Catherine J. Betts

**Affiliations:** ^1^Immunotoxicology, Clinical Pharmacology and Safety Sciences, R&D, AstraZeneca, Cambridge, United Kingdom; ^2^Department of Surgery, University of Cambridge, NIHR Cambridge Biomedical, Cambridge, United Kingdom; ^3^Department of Biochemistry, University of Cambridge, Cambridge, United Kingdom

**Keywords:** immunology and infectious diseases, COVID-19, inflammatory diseases, virus immunity, SARS-CoV-2, inflammation, respiratory disease, comorbidities

## Abstract

Evidence from the global outbreak of SARS-CoV-2 has clearly demonstrated that individuals with pre-existing comorbidities are at a much greater risk of dying from COVID-19. This is of great concern for individuals living with these conditions, and a major challenge for global healthcare systems and biomedical research. Not all comorbidities confer the same risk, however, many affect the function of the immune system, which in turn directly impacts the response to COVID-19. Furthermore, the myriad of drugs prescribed for these comorbidities can also influence the progression of COVID-19 and limit additional treatment options available for COVID-19. Here, we review immune dysfunction in response to SARS-CoV-2 infection and the impact of pre-existing comorbidities on the development of COVID-19. We explore how underlying disease etiologies and common therapies used to treat these conditions exacerbate COVID-19 progression. Moreover, we discuss the long-term challenges associated with the use of both novel and repurposed therapies for the treatment of COVID-19 in patients with pre-existing comorbidities.

## Introduction

The novel severe acute respiratory syndrome coronavirus 2 (SARS-CoV-2), and subsequent SARS-CoV-2 induced coronavirus disease 2019 (COVID-19) has spread on an unprecedented scale. According to the World Health Organization (WHO), as of the 8th July 2020, there have been 11,669,259 cases and 539,906 COVID-19 related deaths worldwide. Evidence from the global outbreak has clearly demonstrated that individuals with pre-existing comorbidities such as hypertension, cardiovascular disease, and diabetes are at a much greater risk of dying from COVID-19 (1, 2). This is of great concern for individuals living with these conditions, and a major challenge for global healthcare systems and biomedical research. Given that comorbidities are associated with high mortality among COVID-19 patients, a better understanding of the biological mechanisms that underpin this risk are needed to enable development of appropriate preventative and therapeutic strategies.

The immune system plays a vital role during COVID-19, and the degree of immune dysfunction correlates with disease severity (3, 4). Severe COVID-19 cases are associated with significant lymphopenia and an overactivated innate immune response resulting in hyperinflammation (5). Many COVID-19 associated comorbidities affect the function of the immune system, which in turn directly impacts the response to COVID-19. Furthermore, the myriad of drugs prescribed for these comorbidities will also influence the progression of COVID-19 and limit additional treatment options available for COVID-19. Here, we review the current SARS-CoV-2 literature and explore how pre-existing comorbidities adversely affect COVID-19 outcome. Furthermore, we discuss the long-term challenges associated with the use of both novel and repurposed therapies for the treatment of COVID-19 in patients with pre-existing comorbidities.

## Human Coronaviruses

In December 2019, several cases of an infectious pneumonia with an unknown etiology emerged in Wuhan Province, China. By January 2020, a novel coronavirus termed SARS-CoV-2 was identified as the cause. The virus spread rapidly and was classified as a pandemic by the WHO on the 11th March 2020. However, this is not the first pathogenic human coronavirus to emerge in the last decade. In 2002, a severe acute respiratory syndrome (SARS) coronavirus (SARS-CoV) with animal to human transmission was reported in Guangdong Province, China (6, 7). Prior to SARS-CoV, four human coronaviruses belonging to the *alpha* and *beta* genera of the *Coronaviridae* family had been identified: HCoV-229E, HCoV-OC43, HCoV-NL63, and HCoV-HKU (8). However, unlike the previously identified coronaviruses, SARS-CoV was phylogenetically distinct (9). Furthermore, rather than causing upper respiratory tract infections with mild common cold symptoms, SARS-CoV caused severe lower respiratory tract infections, resulting in viral pneumonia and risk of developing acute respiratory distress syndrome (ARDS). The SARS-CoV outbreak lasted 8 months, and infected 8,098 individuals across 26 different countries, with a mortality rate of ~10% (10). In 2012, a second novel human coronavirus emerged in Saudi-Arabia and was termed Middle East respiratory syndrome (MERS) coronavirus (MERS-CoV). Similar to SARS-CoV, infection with MERS-CoV can cause fatal pneumonia. To date, the WHO has reported 2,519 MERS-CoV cases, with a mortality rate of ~30% (10).

While the previous two coronavirus outbreaks were relatively well-contained, the unprecedented spread of the current pandemic has demonstrated increased infectivity of SARS-CoV-2. Early genome sequencing from China revealed that the 30 k base-pair viral genome of SARS-CoV-2 shared 79.6% sequence identity with SARS-CoV, whereas a bat coronavirus previously detected in *Rhinolophus affinis* shared 96% sequence identity (11, 12). Whilst MERS-CoV utilizes dipeptidyl peptidase-4 (DDP4) for cell entry, SARS-CoV, and SARS-CoV-2 share the same cell entry receptor; angiotensin converting enzyme II (ACE2) (11, 12). ACE2 is recognized by the S1- subunit of the spike protein and is ubiquitously expressed in the epithelia of the nasal cavity, airway tract and the alveolar space. Notably, reports have shown that the receptor binding domain of SARS-CoV-2 S1 has a higher affinity for ACE2, which may be one contributing factor to the increased viral pathogenesis of SARS-CoV-2 (13, 14). The scale of the ongoing pandemic demonstrates the need for a more comprehensive understanding of the disease, and of the contributing factors such as pre-existing comorbidities, which are proving detrimental for disease severity and outcome.

## Clinical Presentation of COVID-19

**Figure 1:**
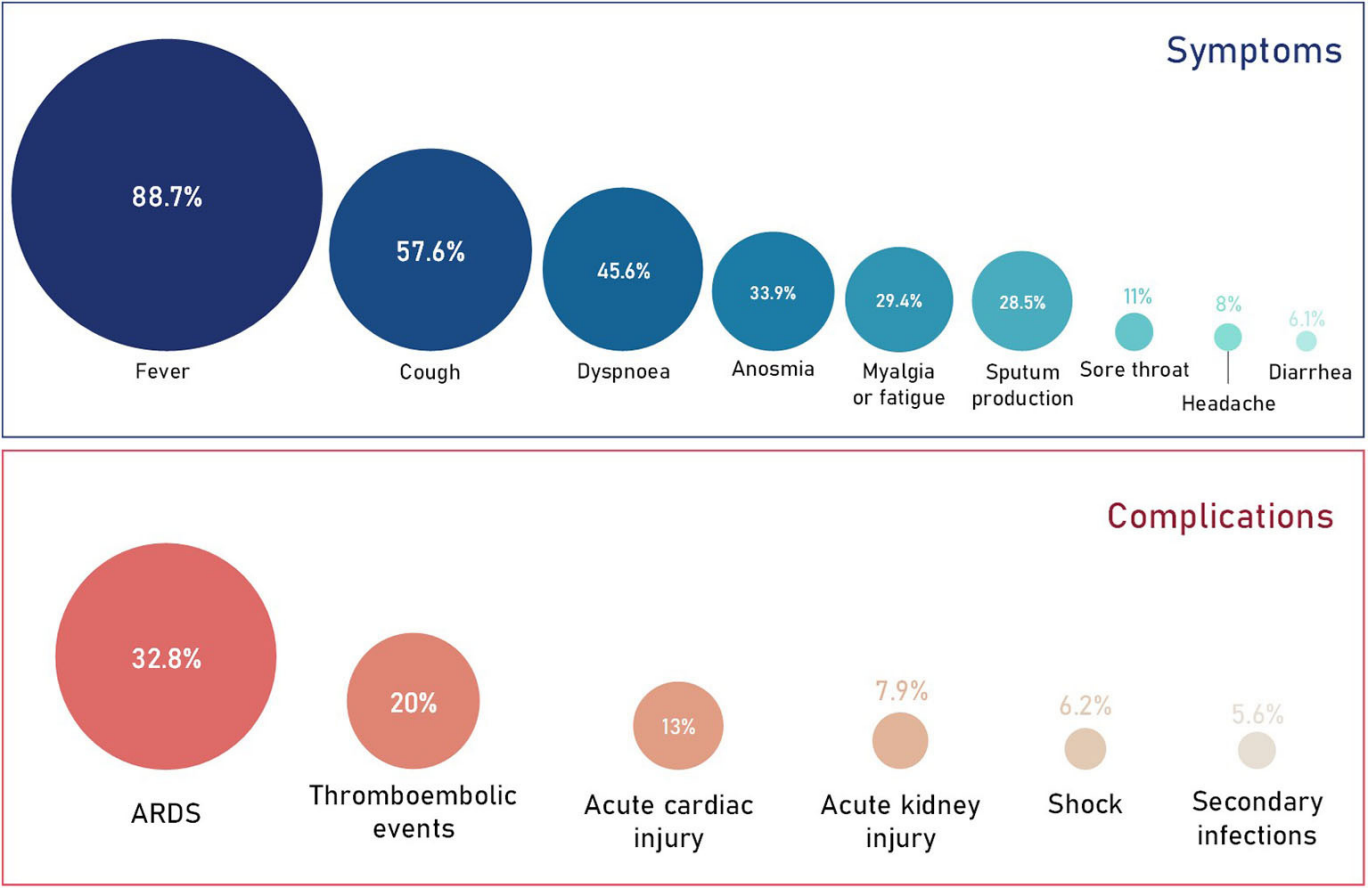
Prevalence of COVID-19 symptoms and complications. Data obtained from a quantitative meta-analysis of 19 studies (19). It's important to note that 87% of all cases analyzed in the meta-analysis were hospitalized cases. Therefore, the proportion of symptoms and complications are representative of this and the overall proportions with regards to all COVID-19 cases will be much lower. The prevalence of anosmia (20) and thromboembolic events (21) were obtained independently from smaller cohorts and may therefore change as more data is published.

Clinical presentation of COVID-19 varies greatly. A meta-analysis of 61 studies from 11 countries (59,254 patients) reported 81.4% of cases as mild, 13.9% as severe and 4.7% as critical (15). Most healthy individuals are asymptomatic or present with mild/moderate respiratory illness (16). The majority of critical cases occur in older (≥60 years) or comorbid individuals (15, 17, 18). COVID-19 symptoms are typical of pathogenic human coronaviruses (Figure 1) (22), with fever and cough reported most commonly, however other common symptoms include dyspnoea, sore throat, sputum production, fatiguem and headache (17, 18, 23). More recently, olfactory dysfunction such as anosmia has been described in COVID-19 patients (20, 24–27). Furthermore, rare gastrointestinal symptoms such as nausea, diarrhea and vomiting have also been described (17, 28, 29). Patients with severe COVID-19 can develop serious and potentially fatal complications such as ARDS, thromboembolic events, septic shock and multiple organ failure (Figure 1). Due to the severity of these complications, many are associated with critical COVID-19 patients who require intensive care (19, 30). Due to underreporting and large inter-country variation, the fatality rate remains unclear. For instance, in Italy the overall case-fatality rate has been reported as 7.2% compared to 2.3% in China (31). However, despite the variation, the evidence clearly demonstrates a higher fatality rate among those who develop severe COVID-19 (32).

### Immune Dysfunction and Disease Severity

**Figure 2:**
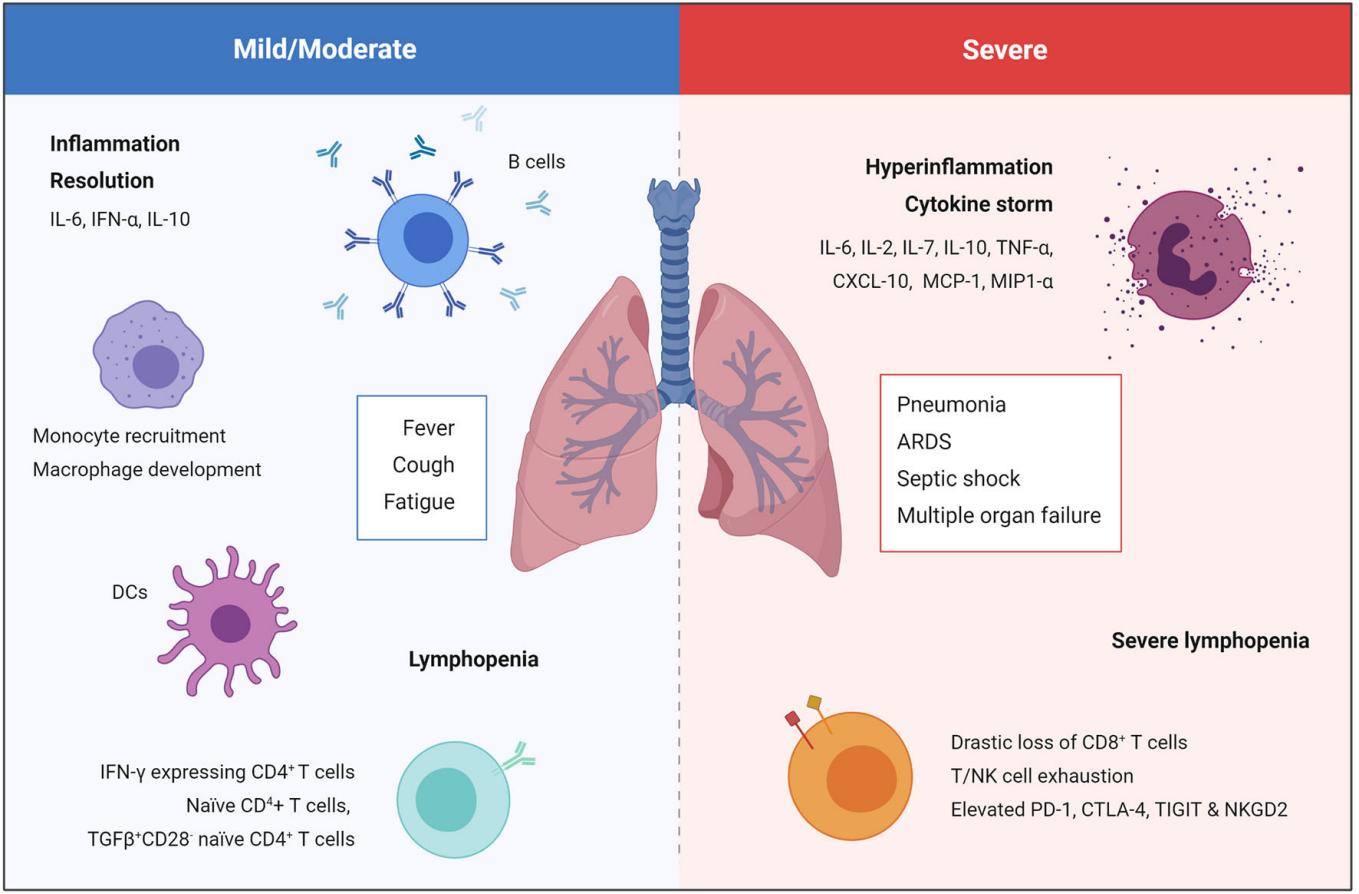
Immune response in mild/moderate and severe COVID-19 cases. Mild/moderate COVID-19 is characterized by local inflammation, the recruitment of monocytes, DCs, NK cells, T and B cells, followed by resolution of the infection and inflammation. Severe COVID-19 is characterized by severe lymphopenia, T cell exhaustion, and systemic hyperinflammation that can cause the development of critical and potentially life-threatening complication such as severe pneumonia, ARDS, septic shock, and multiple organ failure (17, 30, 33).

The immune system plays a vital role during COVID-19, and the degree of immune dysfunction correlates with disease severity (Figure 2) (3, 4). During SARS-CoV-2 infection the immune system becomes activated, resulting in local inflammation, the recruitment of monocytes, dendritic cells (DCs), natural killer (NK), T and B cells. This response may manifest as mild/moderate disease resulting in a fever, cough and fatigue, however this will be followed by resolution of both the infection and inflammation. In severe COVID-19 cases, severe lymphopenia and the accumulation of functionally exhausted T and NK cells result in an inability to mount an effective antiviral immune response to clear SARS-CoV-2 (34, 35). Furthermore, interleukin 6 (IL-6) levels remain elevated over time, and are accompanied by high levels of IL-2, IL-7, IL-10, tumor necrosis factor-α (TNF-α), C-X-C motif chemokine 10 (CXCL-10), monocyte chemoattractant protein-1 (MCP-1), and macrophage inflammatory protein-1α (MIP-1α) resulting in systemic cytokine storm (30). This uncontrolled systemic hyperinflammation can cause the development of critical and potentially life-threatening complications such as severe pneumonia, ARDS, septic shock and multiple organ failure (17, 30, 33). Both lymphopenia and hyperinflammation are being reported in the majority of COVID-19 cases admitted to hospital and is associated with a poor prognosis (4, 32, 36).

More detailed examination has demonstrated negative effects on all lymphocyte subpopulations including CD4^+^ and CD8^+^ T cells, B cells, and NK cells (3). High-dimensional analysis of circulatory immune profiles in mild, moderate and severe COVID-19 patients by mass cytometry revealed that proportions of naïve CD4^+^ T cells, TGFβ^+^CD28^−^ naïve CD4^+^ T cells, DCs, and macrophages are associated with mild cases, whereas a sharp decline in the proportion of CD8^+^ T cells and NK cells was observed in severe cases (3). Interestingly, single-cell RNA sequencing of PBMCs isolated from hospitalized COVID-19 patients revealed a novel population of developing neutrophils, which appeared to be closely related to plasmablasts, in patients that had developed ARDS (37). As this was a small cohort of patients, further studies are needed to assess whether this novel subset of neutrophils plays a role in the development of ARDS and other COVID-19 complications. Functionally, CD8^+^ T cells and NK cells in severe COVID-19 patients exhibited more signs of exhaustion than mild/moderate patients (34, 35). For example, elevated programmed cell death protein-1 (PD-1), cytotoxic T-lymphocyte-associated protein-4 (CTLA-4) and T cell Ig and ITM domain (TIGIT) on CD8^+^ T cells and increased NKG2A on NK cells (34, 35). As exhausted T and NK cells are less able to mount an effective antiviral immune response, it is unsurprising that these subsets are unable to eradicate SARS-CoV-2 and correlate with severe COVID-19 cases.

In addition to T cell changes, humoral immunity against SARS-CoV-2 is starting to come to light. Evidence of SARS-CoV-2 specific antibodies was demonstrated in a study of 173 hospitalized patients. IgG and IgM SARS-CoV2 antibodies were present in 40% of patients within 1 week of onset and 100% by day 15 (38). In another study, most patients developed robust antibody responses between 17 and 23 days, and although delayed, a stronger antibody response was observed in critical patients (39). These findings were also mirrored in a study of 285 COVID-19 patients, who all showed a positive IgG response by day 19, followed by seroconversion to IgM (40). Interestingly, antibody titers were found to be higher among severe COVID-19 patients (40), however the authors acknowledge that interpreting an association between antibody response and disease severity is difficult due to the small sample size of severe and critical patients in their study. A recent study reported low variable plasma antibody titers in convalescent individuals, however they found binding domain specific antibodies with potent anti-viral activity in all individuals (41).

## Impact of Age, Biological Sex and Ethnicity

Older individuals (≥60 years) are more prone to severe COVID-19 and have a higher mortality rate (15, 18, 36, 42). Clinically, older patients have more pronounced immune dysfunction compared to younger patients, as lymphocyte counts are lower and pro-inflammatory cytokine levels higher (43). This is not surprising as aged immune systems are associated with immunosenescence and chronic low-grade inflammation, termed inflammaging (44). Although immunosenescence affects all aspects of the immune system, much of the deterioration in protective viral immunity can be attributed to defective T cell immunity (45). The decline of naïve T cell output due to thymic involution (46) and the accumulation of senescent T cells leads to reduced viral host immunity (47). In mice, CD4^+^ T cells were shown to be crucial against SARS due to their important role in SARS-CoV clearance. This protection was lost in aged mice as senescent CD4^+^ T cells responded poorly to antigen (48, 49). Moreover, in addition to inflammaging, the accumulation of senescent CD8^+^ T cells and B cells with distinct senescence-associated secretory phenotypes (50, 51) in older individuals results in elevated baseline inflammation, further increasing susceptibility to hyperinflammation and cytokine storm upon SARS-CoV-2 infection.

In addition to age, biological sex and ethnicity have also been implicated in COVID-19 outcomes. Although no major sex differences exist when examining absolute number of COVID-19 cases, disease incidence is higher in males when comparing older individuals (≥60 years). Furthermore, initial reports from China suggested a male bias in mortality (23, 36), which has now been reported in 37 out of 38 countries that have reported sex-disaggregated data, revealing a global male case fatality rate of 7.3% compared to 4.4% in females (52, 53). This finding is consistent with data obtained from the previous SARS and MERS epidemic (54–56). The predominant hypothesis to explain these biological sex differences is that estrogen plays a protective role against COVID-19. Following the SARS epidemic, studies in mice demonstrated that ovariectomy or pharmaceutical blocking of estrogen in female mice resulted in elevated immune cell infiltration in the lung and consequently a more severe disease outcome (57). In support of this, researchers in China reported that lower levels of estrogen were associated with more severe COVID-19 cases in women (58). Although the exact molecular mechanisms underpinning how estrogen protects against COVID-19 are yet to be confirmed, the influence of estrogen on aging and immunity, ACE2 levels, and sex-related risk factors for comorbidities have all been suggested (52, 53, 59). Due to these benefits, researchers in the UK have commenced investigations into the effects of hormonal therapies such as the contraceptive pill and hormone replacement therapy, however no data has been published yet. More recently, data has emerged suggesting that Black, Asian and Minority Ethnic (BAME) individuals are at a greater risk of acquiring SARS-CoV-2 and have worse clinical outcomes (60). For instance, in the UK two thirds of COVID-19 fatalities among healthcare workers were BAME individuals (61, 62). Underlying comorbidities, which are more prevalent in BAME individuals, in addition to cultural, behavioral and socio-economic differences have been proposed as possible causes (60). However, more data is needed in order to truly establish whether a relationship between COVID-19 and ethnicity exists.

## Impact of Pre-Existing Comorbidities

**Figure 3:**
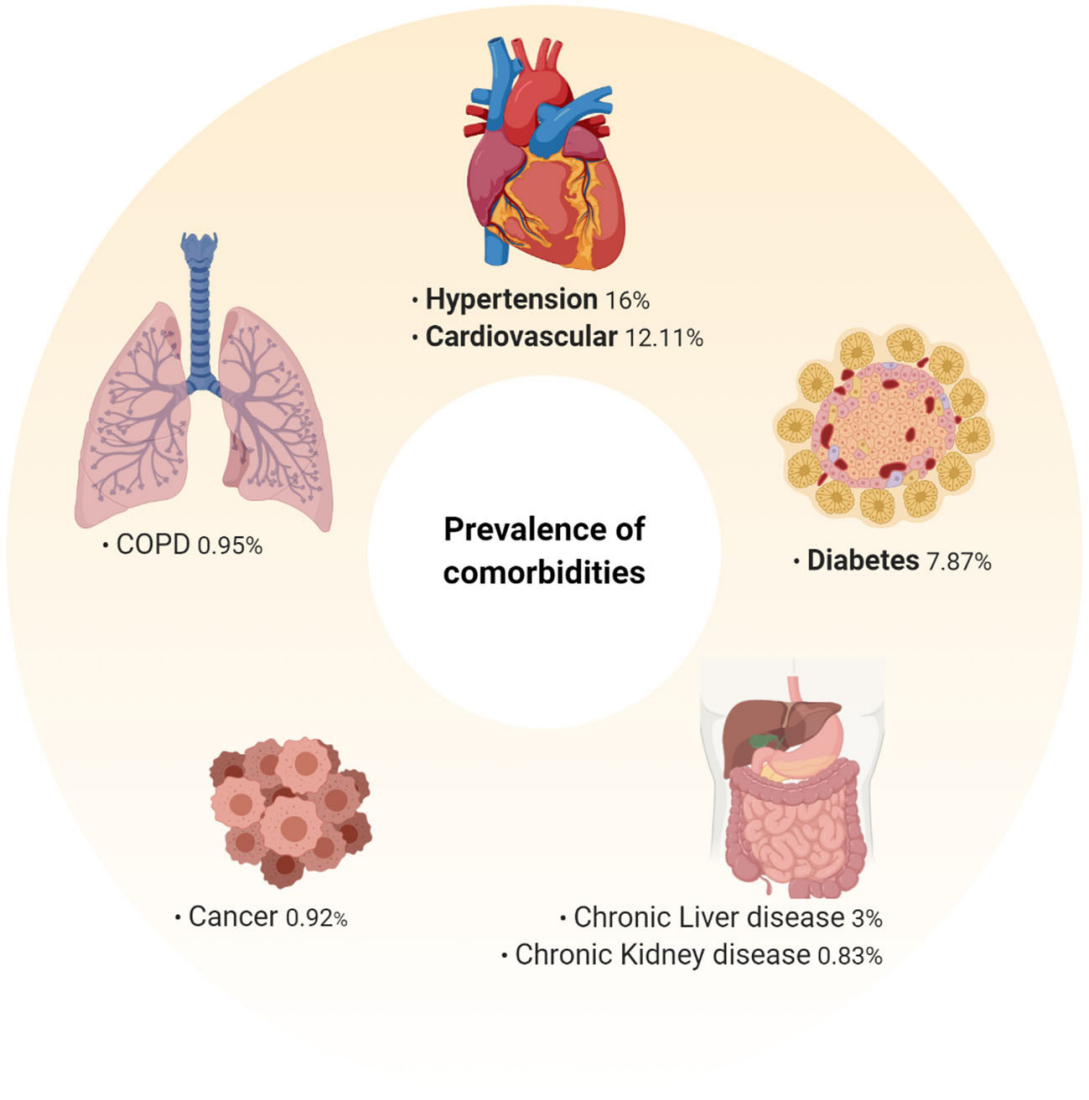
Prevalence of pre-existing comorbidities among hospitalized COVID-19 patients. Prevalence of pre-existing comorbidities determined from an extensive meta-analysis of 76,993 hospitalized COVID-19 patients (1). Hypertension (16%), cardiovascular disease (12.11%), and diabetes (7.87%) were the most prevalent pre-existing co-morbidities. As this data is representative of hospitalized patients, the prevalence of these among mild/moderate cases may be different, and as more data is analyzed these may change.

Evidence from the global outbreak has demonstrated that individuals with pre-existing comorbidities are at a much greater risk of dying from COVID-19 (1, 2). However, a greater understanding of the biological mechanisms that underpin this risk is needed to develop appropriate preventative and therapeutic strategies. Here we review the major comorbidities identified in a number of meta-analyses (Figure 3) (1, 2). After identification, independent searches were conducted in order to comprehensively assess the impact of each comorbidity and its associated therapies on SARS-CoV-2 risk, COVID-19 progression and outcome, and future COVID-19 therapy options.

### Hypertension and Cardiovascular Disease

Hypertension has been repeatedly reported as the highest pre-existing comorbidity in COVID-19 patients (1, 2, 17, 63–65). Retrospective analysis revealed that patients with hypertension have an increased risk for severe infection and mortality (65, 66). However, whether hypertension itself or the use of hypertensive therapies are responsible for these statistics is currently unknown. Hypertensive patients are commonly treated with renin angiotensin system inhibitors, such as ACE inhibitors (ACEI) and angiotensin-receptor blockers (ARB). As ACEI and ARB can significantly increase ACE2 expression (67), many speculate they are responsible for the increased risk to hypertensive patients (68). Conversely, a retrospective review of 417 hospitalized COVID-19 patients, of which 12.23% had underlying hypertensions, indicated that ACEI and ARB may be protective effect against COVID-19, as the percentage of severe cases were lower in patients treated with ACEI/ARB (23.5%) when compared to those treated with other anti-hypertensive treatments such as calcium channel blockers, β-blockers, and diuretics (48%) (69). However, patients treated with non-ACI/ARB were also found to have a higher incidence of additional comorbidities (69), which may have been responsible for the development of severe disease. Due to the conflicting evidence and opinions among the scientific community, it remains unclear whether treatment with ACEI/ARB has a positive or negative impact on COVID-19 progression, however many are continuing to examine this.

Cardiovascular disease has also been highly reported among COVID-19 patients and is associated with an increased mortality rate (17, 63, 70). Furthermore, cardiovascular complications such as thromboembolic events, myocarditis, acute coronary syndrome, arrythmia, cardiogenic shock and heat failure, have been documented in COVID-19 patients without prior cardiovascular disease (71), demonstrating a significant impact of SARS-CoV-2 infection on the heart. In a case series of 187 COVID-19 patients, those with underlying cardiovascular disease had a mortality rate of 37.5%, which was further increased to 69.44% in a subset of patients who had both underlying cardiovascular disease and elevated troponin T levels, indicative of myocardial injury (70). Two possible explanations for the increased prevalence and mortality among patients with comorbid cardiovascular disease have been proposed. Firstly, cardiovascular disease is commonly treated with renin angiotensin system inhibitors as described above (72, 73), and secondly, ACE2 is highly expressed in the heart (74). A recent study analyzing the cellular distribution of ACE2 in human heart tissue obtained from COVID-19 patients identified that ACE2 was highly expressed in pericytes, cardiomyocytes and fibroblasts (75). Furthermore, CD209, an additional binding receptor for SARS-CoV, was specifically expressed in macrophages (75). Due to the increased presence of macrophages in cardiovascular disease, the authors speculate that CD209^+^ macrophages may enhance viral entry into the human heart. Collectively, this study indicated an intrinsic susceptibility to SARS-CoV-2 infection in the heart, which could explain the high susceptibility to COVID-19 among cardiovascular disease patients and the higher incidence of acute cardiac injury in non-cardiovascular disease patients.

According to numerous reports severe COVID-19 patients are at heightened risk of thromboembolic events, with 20–30% of critically ill COVID-19 patients reported to have developed thromboembolic complications (21, 76–79). Systemic inflammation and subsequent activation of coagulation are both contributing factors to this increased risk (79, 80). Coagulation abnormalities have been reported throughout the COVID-19 pandemic and the term COVID-19-associated coagulopathy (CAC) has been used to describe patients displaying coagulation changes (78). Elevated levels of prothrombin, fibrinogen and D-dimer, in addition to elevated inflammatory markers such as C-reactive protein (CRP) and IL-6, are markers of CAC (78). In particular, increased D-dimer levels highly correlate with disease severity, as elevated D-dimer presenting at admission or over time are associated with increase mortality in COVID-19 patients (81). Due to the high incidence of thromboembolic events, the use of thromboprophylaxis for patients admitted to hospital with severe COVID-19 has been suggested (76, 78), and CAC should be monitored carefully in all hospitalized COVID-19 patient, particularly those with pre-existing risk of thromboembolic events.

### Diabetes and Obesity

Diabetes is the third most prevalent underlying comorbidity in COVID-19 patients (2, 17, 63, 82, 83). Type 2 diabetes is a multifactorial disease characterized by chronic inflammation and impaired metabolism and has become an increasing risk to human health. Diabetic individuals have an increased susceptibility to infection (84), and are at a high risk of developing multiple comorbidities such as cardiovascular disease (83). One study on COVID-19 found that diabetic patients were more likely to develop pneumonia and were responsible for 11.7% of severe cases, but only 4% of mild/moderate cases (85). There are a number of reasons as to why people with diabetes are more likely to develop severe COVID-19. Firstly, chronic inflammation in diabetic patients increases their susceptibility to hyperinflammation and the development of cytokine storm. This has been already reported in COVID-19 patients, as IL-6 and CRP levels were found to be significantly higher in diabetic patients (83). Secondly, it is well-documented that hyperglycaemia can impair the immune response, increase oxidative stress and is associated with the onset of premature senescence (86, 87). Consequently, diabetic patients that are unable to control their blood glucose levels may have an even greater vulnerability to severe disease. Finally, in addition to disease etiology, the treatment of diabetes may also impact COVID-19 development. As previously discussed for hypertension and cardiovascular disease, the use of renin angiotensin system inhibitors may increase susceptibility to SARS-CoV-2 infection (88, 89). Furthermore, DPP4 inhibitors commonly used to treat diabetes have an anti-inflammatory effect, resulting in reduced macrophage infiltration, which could impair the innate immune response during COVID-19 (90).

Obesity is associated with most of the common COVID-19 comorbidities such as hypertension, cardiovascular disease and diabetes (91, 92). The global prevalence of obesity varies greatly, for example obesity is more common in the United States and Europe than it is in Asian countries (93). Consequently, COVID-19 severity and mortality rates may also vary as a result of this. One study reported a higher BMI in patients with severe infection, and when comparing survivors vs. non-survivors, it was reported that 88.2% of non-survivors had a BMI above 25 kg/m^2^, which was a significantly higher proportion than survivors (94). However, the cohort for this study was small (*n* = 30) and further retrospective analysis of existing studies is needed to clarify the impact of obesity on COVID-19. Following the previous H1N1 pandemic, retrospective analyses reported that obesity was associated with increased risk of severe infection and mortality (95), which is in line with the increased risk of infection in obese individuals (96). In addition to increased risk of comorbidities, obesity is also linked to an impaired immune response (97), with evidence of impaired antibody (98) and T cell (99) responses. Furthermore, expression of ACE2 is upregulated in adipocytes of obese individuals and therefore may act as a potential target for SARS-CoV-2 (100).

### Cancer

The incidence of cancer as a COVID-19 comorbidity has been low. In an extensive meta-analysis of 76,993 patients, malignancy accounted for just 0.92% of comorbidities reported (1). However, a nationwide analysis in China reported that 1.1% of COVID-19 cases had active cancer, and these patients had a higher proportion of serious events in comparison to individuals without cancer (101). At this current time, it is unclear whether cancer patients are at high risk of COVID-19 due to an immunocompromised state linked to certain cancer therapies. In one particular study, patients that underwent recent chemotherapy or surgery had a higher risk of clinically severe events than those who did not. However, a limited number of just 19 patients were included in this analysis (102), demonstrating the need for more thorough research.

Furthermore, not all cancer patients should be considered equally immunocompromised. Cancer patients treated with immuno-oncology therapies such as immune checkpoint inhibitors (ICI) could be more immunocompetent than patients undergoing chemotherapy (103). Nonetheless, there are two major concern associated with the use of immuno-oncology therapies during COVID-19. Firstly, there is potential overlap between COVID-19 interstitial pneumonia and possible pneumological toxicity from anti-PD-1/PDL-1 agents, which can be fatal. Although this is a rare immune-related adverse event, it has been reported in 2.5–5% of patients treated with anti-PD-1/PDL-1 monotherapy, and 7–10% of patients treated with anti-PD-1/anti-CTLA-4 combination therapy (104, 105). Secondly, there is a risk of cytokine release syndrome associated with T cell-engaging immunotherapy, such as chimeric antigen receptor (CAR) T cells. Given that cytokine storm has been linked to a negative outcome in COVID-19 due to the development of ARDS and multiple organ failure, ICI and CAR-T cell therapies may exacerbate this hyperinflammatory state and increase mortality in these patients (103). Overall, despite the lower incidence of cancer among COVID-19 patients, those being treated with immunocompromising therapies such as chemotherapy, and those susceptible to immune-related adverse events in response to immuno-oncology therapies should be monitored carefully.

### Respiratory Diseases

As infection with SARS-CoV-2 results in an acute respiratory disease that can progress to ARDS, respiratory failure and potentially even death, it is reasonable to speculate that patients with pre-existing respiratory disease would be at increased risk of severe COVID-19. Surprisingly, this risk is not as striking as one might anticipate, as the prevalence of asthma was just 0.90% in a study of 548 COVID-19 patients in China (106). Furthermore, the incidence of chronic obstructive pulmonary disease (COPD) among hospitalized COVID-19 patients was reported to be just 0.95% in a meta-analysis of 76,993 patients (1). Conversely, the Center For Disease Control and Prevention (CDC) recently released data of US hospitalizations indicating that COPD is present in 9.2% of patients (107). A possible explanation for this global variation could be due to differences in therapeutics agents and disease management. For instance, a global survey assessing the severity and control of 7,786 asthmatic adults worldwide published that individuals in Japan and Asia-Pacific regions reported less severe disease than those in Europe and the United States (108).

### Liver and Kidney Disease

The prevalence of chronic liver disease in COVID-19 patients is estimated to be 3% (19, 109). However, liver damage has been repeatedly reported as a common complication in response to COVID-19 (30, 36, 109). In a study of 1,100 patients in China, elevated levels of the liver enzyme aspartate aminotransferase (AST) were reported in 18% of mild/moderate COVID-19 patients, and 56% of severe COVID-19 patients (36). The same study also reported elevated levels of alanine aminotransferase (ALT) in 20% of mild/moderate COVID-19 patients and 28% of severe COVID-19 patients (36). Consequently, it has been proposed that liver damage associated with severe COVID-19 patients is due to dysregulated innate immunity against SARS-CoV-2, or hepatoxicity in response to treatments, rather than pre-existing liver disease.

Chronic kidney disease is associated with an increased risk of pneumonia, and elevated mortality from infectious diseases has been reported in patients with end stage renal disease (110). In an extensive meta-analysis, chronic kidney disease accounted for 0.83% of comorbidities in COVID-19 patients (1). While the prevalence of chronic kidney disease among COVID-19 patients is low, those with pre-existing kidney disease have been associated with severe disease and increased mortalities (111). ACE2 expression in the kidney is elevated in chronic kidney disease (112). However, elevated ACE2 expression in the kidney does not appear to correlate with increased susceptibility to SARS-CoV-2 in the same way as it does in the heart. Nonetheless, chronic kidney disease is associated with persistent, low-grade inflammation which could exacerbate COVID-19 symptoms. Several factors contribute to this inflammation such as elevated cytokines including IL-6 and CRP, oxidative stress and impaired metabolism (113). Therefore, the underlying pathogenesis of chronic kidney disease may increase vulnerability to hyperinflammation and cytokine storm upon SARS-CoV-2 infection, resulting in severe COVID-19.

### Autoimmune Diseases

Autoimmune diseases are conditions characterized by inappropriate immune activation and destruction of healthy cells. Lymphopenia is common among autoimmune diseases such as type 1 diabetes, rheumatoid arthritis and systemic erythematosus lupus (114), and since lymphopenia is regarded as a major risk factor for developing severe COVID-19, individuals living with an autoimmune condition may be perceived as high risk. However, unlike other comorbidities mentioned above, autoimmune diseases have not been reported as a risk factor in the current meta-analyses. One possible explanation could be that autoimmune diseases are often treated with drugs designed to restrict immune activation, many of which are now being repurposed for COVID-19 and will be discussed below. Furthermore, potential alterations in patient behaviors in order to shield themselves from infection could influence reporting of autoimmunity as a risk factor.

## Considerations for COVID-19 Therapies

**Figure 4:**
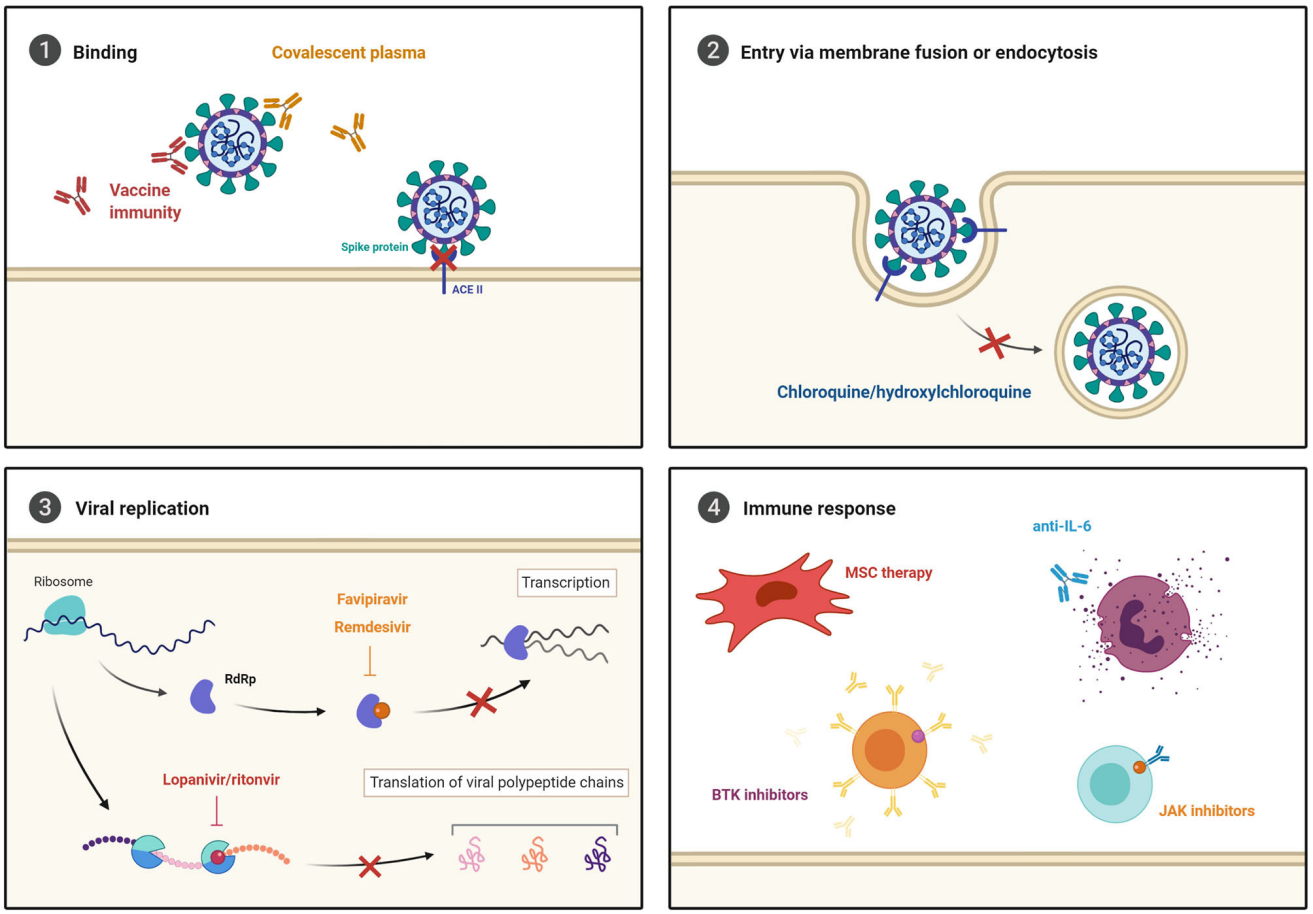
Current COVID-19 treatment strategies. (1) Novel vaccines and convalescent plasma are being investigated to prevent viral entry. Broad-spectrum antiviral agents that inhibit (2) cell entry via endocytosis, and (3) viral replication through RNA-dependent RNA polymerase (RdRP) and protease inhibition are being repurposed to test efficacy against SARS-CoV-2. Furthermore, (4) immune modulating and anti-inflammatory treatments are being explored to prevent the development of severe COVID-19 development.

As the pandemic continues to accelerate globally, so does the race to develop an effective therapy to protect against and treat COVID-19 (Figure 4). However, robust efficacy and safety assessments are needed, particularly with regards to their use in comorbid patients, as reviewed below.

### Vaccines

The SARS-CoV-2 genome and spike protein structure was discovered very rapidly (115), enabling focussed development of both RNA- and protein-based vaccines. However, vaccine development is a challenging and time-consuming process. Following the SARS-CoV epidemic, several vaccines were developed and assessed using animal models. Vaccination with live virus was shown to cause complications in mice such as lung damage (116, 117), and although recombinant spike protein based vaccines were able to protect animals from SARS-CoV challenge, they were ineffective at inducing sterilizing immunity (118). Other vaccines with inactivated SARS-CoV and MERS-CoV have demonstrated reduced viral titers, reduced morbidity and greater survival in animal models (119, 120). However, the rapid eradication of SARS-CoV reduced demand for development. Some MERS-CoV vaccines are currently in pre-clinical and clinical development (121), however as MERS-CoV is less closely related, it is unlikely that these will cross-protect against SARS-CoV-2. Learnings from previous coronavirus outbreaks indicate that antibody responses are not particularly long-lived, with SARS-CoV antibodies lasting on average just 2 years (122). This apparent lack of long-term protection exacerbates the need for an effective vaccine or vaccine programme to protect against potential recurrent seasonal SARS-CoV-2 infections.

On the 6th July 2020, the WHO reported 160 candidate SARS-CoV-2 vaccines under development worldwide (123). Some of the most promising include an mRNA vaccine; mRNA-1273 (124), a DNA plasmid vaccine (125), and an adenovirus vaccine; ChAdOx1 nCoV-19 (126). Although clinical evaluation is underway, outcomes are not available yet. Furthermore, vaccine efficacy depends upon an individual's ability to mount a strong immune response against it. Consequently, vaccine regimens that grant protection in healthy individuals may not be adequate for older individuals and those with comorbid conditions who have increased immunosenescence (127). For influenza, the use of adjuvant (128) and high dose vaccines (129, 130) have been developed and implemented to increase vaccine immunogenicity for older and high-risk comorbid adults. Therefore, similar measures are likely to be needed to fine tune a SARS-CoV-2 vaccine once it becomes available. In the interim, rolling out a SARS-CoV-2 vaccine with proven efficacy in healthy individuals could result in herd immunity, preventing transmission to those more vulnerable and offer indirect protection.

### Convalescent Plasma Therapy

Another therapeutic approach intended to prevent COVID-19 is convalescent plasma therapy. Previous use during the SARS (131) and MERS (132) epidemics, and H1N1 pandemic (133), demonstrated reasonable efficacy and safety. Evidence from those outbreaks revealed that convalescent plasma contained neutralizing antibodies (134), and a meta-analysis from 32 SARS-CoV and influenza studies, reported a significant reduction in mortality following convalescent plasma therapy (135). Several convalescent plasma studies for SARS-CoV-2 have been reported. In China, a small pilot study was conducted to test convalescent plasma collected from 40 recently recovered patients on 10 severe COVID-19 patients, four of which had underlying conditions including hypertension and cardiovascular disease (136). From the 40 patients, 39 had high neutralizing antibody titers of ≥1:640. No serious adverse events were reported following transfusion, and all patients experienced improved symptoms within 1 to 3 days post-transfusion. However, there are many caveats to this study, the very small sample size and the use of concomitant treatments mean it is not possible to ascertain if clinical improvements were due to convalescent plasma. Consequently, additional large scale, controlled, randomized trials are needed and are currently underway.

Despite no adverse events being reported in the small sample sizes currently being tested for COVID-19, plasma transfusions are not without risk. As with any transfusion, there is a risk of transfusion transmitted infections, albeit small (137). Perhaps of more concern are the non-infectious risks such as allergic reactions, transfusion related acute lung injury characterized by acute hypoxemia and pulmonary oedema, and transfusion associated circulatory overload, characterized by hypertension, tachycardia, tachypnoea and dyspnea (138). These are of particular concern for severe COVID-19 patients with extensive lung damage, and for those with pre-existing hypertension, cardiovascular disease or renal failure (138, 139). Due to the increased prevalence of comorbidities among COVID-19 patients, those predisposed to transfusion-related adverse events will need to be carefully evaluated. As transfusion volume and rate have both been identified as risk factors for transfusion associated circulatory overload (140), COVID-19 trials should examine these parameters to ensure safety in comorbid patients.

## Challenges to Overcome With Repurposed Drugs

While many scientists attempt to develop novel therapies to prevent COVID-19, others are focusing their attention on repurposing drugs that are already on the market (Figure 4).

### Chloroquine and Hydroxychloroquine

At present, the most contested repurposed drug is chloroquine and its analog hydroxychloroquine. Following the 2002 SARS-CoV epidemic, researchers found that *in vitro* treatment of chloroquine could increase endosomal pH and impair terminal glycosylation of ACE2 receptors on the cell surface, therefore inhibiting SARS-CoV-ACE2 interactions and preventing virus entry (141, 142). Similarly, after the 2012 MERS-CoV epidemic, researchers demonstrated that chloroquine could inhibit *in vitro* replication of MERS-CoV in well-established cell lines (143) and primary mature antigen presenting cells (144). However, as SARS-CoV and MERS-CoV resolved relatively quickly, continued assessment of chloroquine remained limited. Furthermore, although *in vitro* treatment can inhibit SARS-CoV-2 (145), the use of chloroquine and hydroxychloroquine for COVID-19 in the clinic is highly controversial. A series of early clinical trials conducted in China reported apparent efficacy of chloroquine phosphate in treating COVID-19 (146). This was followed by a small, non-randomized study of just 36 patients from France, which demonstrated a clinical benefit from the use of hydroxychloroquine, but lacked an appropriate control group and a long-term follow-up (147). However, an observational study of 1,376 patients treated with hydroxychloroquine report no sign of efficacy for COVID-19 (148). This was further confirmed by results from the large Randomized Evaluation of COVID-19 Therapy (RECOVERY) trial that examined 1,542 hospitalized COVID-19 patients treated with hydroxychloroquine and reported no significant difference in mortality when compared to 3,132 standard of care patients (149, 150).

### Broad-Spectrum Antiviral Agents

#### Lopinavir/Ritonavir

Lopinavir is a protease inhibitor often formulated with low-dose ritonavir and traditionally used to treat HIV patients (151). Lopinavir/ritonavir was previously assessed in a small, non-randomized study conducted in Hong Kong during the SARS-CoV epidemic. This study demonstrated that lopinavir/ritonavir treatment resulted in reduced viral load and milder disease, with less recurrence of fever, diarrhea, and improved chest radiographs when compared with those treated with ribavirin, an alternative antiviral (152). During the current outbreak, two independent case studies of COVID-19 patients hospitalized in South Korea, reported a reduction in viral load and subsequent recovery of patients following the administration of lopinavir/ritonavir (153, 154). However, a randomized control study in China of 199 severe COVID-19 patients demonstrated no difference in mortality, or in the amount of viral RNA detected (155). Furthermore, recent findings from the RECOVERY trial comparing 1,596 patients randomized to lopinavir/ritonavir to 3,376 standard of care patients concluded no clinical benefit from the use of lopinavir/ritonavir (149, 156). Moreover, protease inhibitors such as lopinavir/ritonavir have been associated with hepatotoxicity during HIV treatment (157, 158), and drug-to-drug interaction (DDI) with certain statins, for example rosuvastatin, can increase the risk of myopathy (159, 160). Therefore, administration to COVID-19 patients with pre-existing liver disease and those being treated with statins could prove detrimental. Like other members of the protease inhibitor class, lopinavir/ritonavir has also been associated with metabolic changes that can result in hyperglycaemia (161), hyperlipidaemia (162), and insulin resistance (163). Due to the lack of efficacy and increased risk of toxicity to certain COVID-19 patients lopinavir/ritonavir could be prove more harmful to patients and should therefore be avoided.

#### Oseltamivir and Favipiravir

Oseltamivir and favipiravir, *two* antiviral treatments traditionally used to treat influenza, have also been examined for use in COVID-19. Oseltamivir is a neuraminidase inhibitor, which has been extensively used as a prophylactic against influenza. In a small, single-center study of 138 patients with SARS-CoV-2 pneumonia in China, 89.9% of patients received oseltamivir alongside anti-bacterial drugs such as moxifloxacin, ceftriaxone, azithromycin, and glucocorticoid therapy (18). The study concluded no effective outcomes based on oseltamivir (18). However, the small study size, lack of appropriate control and the fact that many patients remained hospitalized at the time of publications limits full interpretations. Favipiravir is an antiviral with potent inhibitory activity against viral RNA-dependent RNA polymerase. Experimentally, favipiravir demonstrated effective SARS-CoV-2 inhibition in Vero E6 cells (145). Furthermore, a small, open-label, non-randomized comparative study of 80 patients in China, compared clinical outcomes of patients treated with favipiravir and lopinavir/ritonavir (164). The median time until viral clearance was 4 days with favipiravir compared to 11 days with lopinavir/ritonavir. At day 14, CT scans of the chest from patients treated with favipiravir demonstrated significant improvements. Adverse events occurred in 11% of favipiravir treated patients compared to 55% of lopinavir/ritonavir treated patients (164). However, despite initial promise more robust clinical data is needed in order to establish the efficacy and safety of favipiravir as a treatment of COVID-19.

#### Remdesivir

Another antiviral agent being examined for use in COVID-19 is remdesivir. When metabolized into its active form, remdesivir inhibits viral RNA polymerases, causing a decrease in viral RNA production. Remdesivir has been shown to inhibit SARS-CoV and MERS-CoV in human airway epithelial *in vitro* models (165, 166), and in combination with interferon beta, remdesivir has been shown to be superior to lopinavir/ritonavir in a MERS-CoV mouse model (167). Despite some initial controversial findings (168), results from more robust clinical trials have demonstrated reasonable efficacy. For instance, preliminary results from the National Institute of Allergy and Infectious Diseases Adaptive COVID-19 Treatment Trial involving 1,063 patients, demonstrated that remdesivir accelerated recovered by 31% compared to placebo (169). Furthermore, recent results from the Phase 3 SIMPLE trial investigating the use of remdesivir in patients with moderate COVID-19 showed that patients receiving remdesivir treatment were 65% more likely to have improved clinically by day 11 than standard of care patients (170, 171). Due to the success of these two trials, the use of remdesivir has been approved by the FDA, EMA, UK, and Japan as a treatment for COVID-19. Despite proven efficacy, adverse events in response to remdesivir have been reported in 60% of patients of which 12% were severe (168). These included septic shock, multiple organ dysfunction syndrome, acute kidney injury and hypotension. Therefore, the use of remdesivir in comorbid patients with increased susceptible to these adverse events requires further evaluation.

### Anti-inflammatory Treatments

As severe COVID-19 cases are characterized by hyperinflammation, the use of immune modulating and anti-inflammatory treatments to prevent severe lung injury and disease progression are being explored.

#### Mesenchymal Stem Cell (MSC) Therapy

Due to their immunomodulatory properties, MSCs are being clinically assessed to treat inflammatory conditions such as systemic lupus erythematosus (172) and graft vs. host disease following allogeneic haemopoietic stem-cell transplantation (173). Consequently, a pilot study was initiated to investigate the potential therapeutic benefit of MSCs for COVID-19 infected patients in China. The study involved just 10 COVID-19 patients who were monitored for 14 days post-MSC injection (174). MSC treatment was well-tolerated and no adverse events occurred during treatment. Furthermore, virtually all clinical symptoms subsided, with 3 patients being discharged 10 days post-MSC injection (174). Mass cytometry of PBMCs revealed that peripheral lymphocytes, regulatory CD14^+^CD11c^+^CD11b^mid^ DCs and IL-10 increased. Whereas, CRP, TNF-α and overactivated CXCR3^+^ CD4^+^/CD8^+^/NK cells decreased 3 to 6 days following injection compared to placebo group. MSCs were shown to be ACE2 negative, meaning they were immune to SARS-CoV-2 infection (174). Although this pilot study shows promise, more robust clinical data is required to validate therapeutic benefit and safety. Moreover, the use of MSCs would require clinical grade MSC production and may not be a plausible solution for many healthcare systems.

#### Anti-IL-6

IL-6 is considered the key cytokine responsible for the induction of cytokine storm during SARS-CoV, MERS-CoV and SARS-Cov-2 infections (30, 175, 176). Consequently, the recombinant humanized anti-human IL-6 receptor monoclonal antibody tocilizumab, currently used to treat rheumatoid arthritis (RA), has been examined for use during COVID-19. Tocilizumab first demonstrated effectiveness in a small retrospective study of 20 patients with severe COVID-19 pneumonia in China (177). Oxygen intake was reduced, and improved symptoms occurred in 75% of patients. Lymphocyte levels returned to normal in 56% of patients and CRP levels reduced significantly in 84.2% of patients (177). Several case studies have also demonstrated rapid clinical improvements following tocilizumab (178–180). Although these initial studies are promising, the full clinical trial data is still unavailable. Furthermore, although safety profiles for tocilizumab are well-established for intermittent use in RA, the safety of tocilizumab when used in combination with antiviral agents and other comorbid therapies has not been established.

#### Kinase Inhibitors

Other anti-inflammatory treatments now being tested clinically for their use against COVID-19 are Janus kinase (JAK) and Bruton's tyrosine kinase (BTK) inhibitors. The JAK-signal transducer and activator of transcription (JAK/STAT) pathway mediates the signal transduction of numerous cytokines in a number of immune cells such as T cell, NK cells, and DCs (181). Consequently, JAK inhibitors have emerged as effective treatments for many autoimmune and immune-mediated disease. At present, a number of JAK inhibitors such as ruxolitinib (182), baricitinib (183), and fedratinib (184), are being assessed as a potential treatment for COVID-19. Preliminary results from a pilot study evaluating 88 hospitalized patients, 20 of which were treated with baricitinib, demonstrated significant reductions in serum IL-6, IL-1β, and TNF-α, as well as a rapid recovery of circulatory T and B cell frequencies following baricitinib treatment (185). Consequently, a Phase 3 Adaptive COVID-19 Treatment Trial (ATTC-2) has now been established in order to evaluation the use of baricitinib in combination with remdesivir compared to remdesivir alone (186). However, despite promising initial findings, as JAK inhibitors block a wide range of cytokines including IFN-α, which is crucial during early innate immunity in response to viral infections, the impact of this on viral clearance needs to be evaluated. Furthermore, ruxolitinib and baricitinib have both been associated with increased weight gain, cholesterol and albumin levels (187, 188). Although no causal association has been reported yet, the issue should not be dismissed and COVID-19 patients with metabolic and cardiovascular comorbidities should be carefully considered before use.

Bruton's tyrosine kinase (BTK) is a key regulator of cell surface receptors expressed primarily in B cells, but also in monocytes/macrophages and neutrophils (189). Currently, BTK inhibitors are used to treat various B cell malignancies and chronic graft vs. host diseases (190). As BTK can regulate IL-6, TNF-α, and MCP-1, BTK inhibitors are being tested in combination with CAR-T cells (191), to alleviate cytokine release syndrome. Furthermore, in chronic lymphocytic leukemia, BTK inhibitors have been shown to increase CD4^+^ and CD8^+^ T cell, and significantly downregulate PD-1 and CTLA-4 (192), highlighting a potential reversal of T cell exhaustion, which could be beneficial in COVID-19. Consequently, clinical trials to assess the use of BTK inhibitors against COVID-19 are underway.

#### Corticosteroids

The use of corticosteroids to treat COVID-19 remained largely uncertain until recently. Although individuals being treated with long term corticosteroid were instructed to continue with their medication, the use of corticosteroids specifically to treat COVID-19 was not recommended (193). This was largely due to the unknown impact of immune suppression on viral clearance and potential adverse outcomes. However, preliminary data from the RECOVERY trial evaluating 2,104 patients randomly allocated to receive dexamethasone has proven significant clinical improvements (194). Dexamethasone is a steroid used to reduce inflammation in a myriad of inflammatory conditions and now reported to reduce COVID-19 related deaths among patients receiving respiratory support by one-third (194). In response to these findings the demand for dexamethasone to treat the most critical COVID-19 patients has surged globally (195). Whilst these findings are exciting, safety data detailing potential adverse events and the impact of co-medications, such as non-steroidal anti-inflammatory drugs, has not yet been reported and thus dexamethasone should still be considered carefully prior to administration.

## Summary

Patients with pre-existing comorbidities are at a greater risk of dying from COVID-19. However, not all comorbidities confer the same risk. By exploring the underlying disease etiologies and common therapies used to treat these conditions, we have discussed their impact on COVID-19. Comorbidities closely associated with age, chronic inflammation and dysregulated metabolism such as hypertension, cardiovascular disease, and diabetes are the most prevalent comorbidities. However, many of these comorbidities are strongly associated with each other. Consequently, many patients will have multiple comorbidities and therefore while we have discussed these individually, the reality is that a combination of factors will be at play. Furthermore, as multiple drug use is inevitable for patients with pre-existing comorbidities, the impact of overlaying drugs on an already compromised state and the possibility of DDI leading to adverse events needs to be carefully considered. As the scale of this pandemic continues to accelerate globally, we hope this review provides healthcare professionals and biomedical researchers with a more comprehensive understanding of the impact of pre-existing comorbidities on COVID-19 development and treatment.

## Author's Note

The authors LC and MC are fellows of the AstraZeneca postdoc programme. Figures were created with BioRender.com.

## Author Contributions

LC and MC wrote the first draft of the article. SB, MM, JW, and CB reviewed and edited the article before submission. All authors contributed extensively to the discussion of the content and researched data for the article.

## Conflict of Interest

All authors are employees of AstraZeneca.
